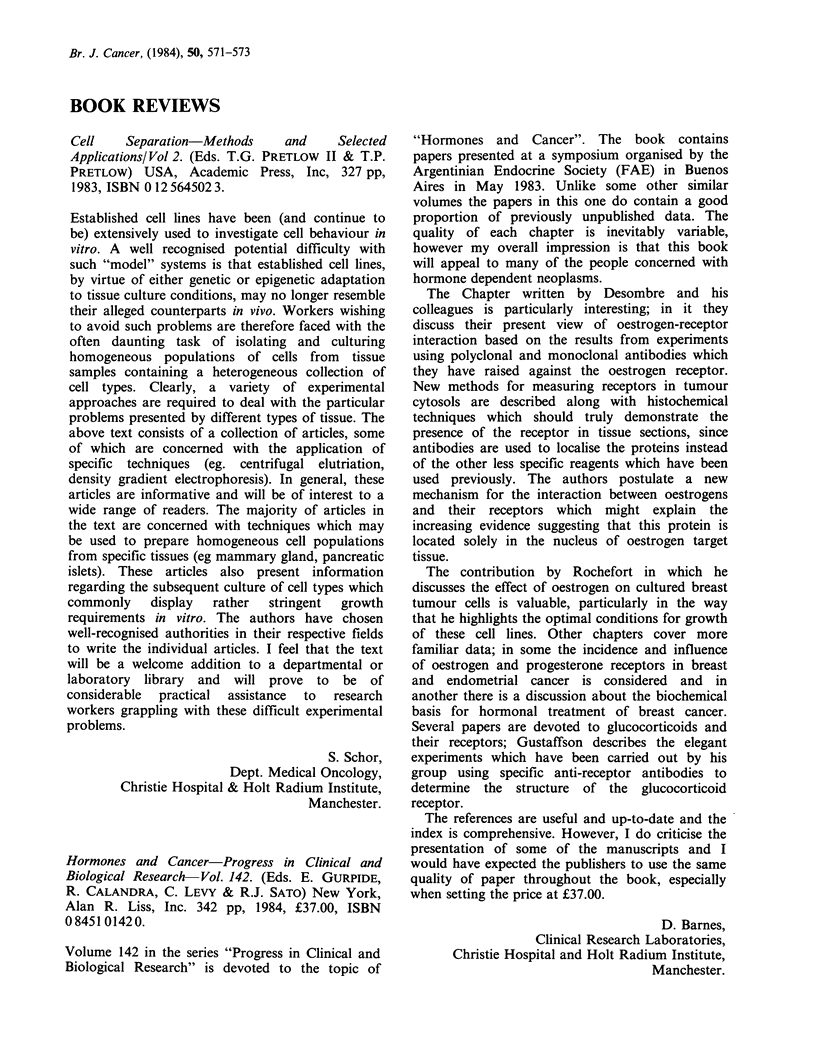# Cell Separation—Methods and Selected Applications/Vol 2

**Published:** 1984-10

**Authors:** S. Schor


*Br. J. Cancer*, (1984), **50**, 571–573

### BOOK REVIEWS

*Cell Separation—Methods and Selected Applications/Vol 2.* (Eds. T.G. Pretlow II & T.P. Pretlow) USA, Academic Press, Inc, 327 pp, 1983, ISBN 0 12 564502 3.

Established cell lines have been (and continue to be) extensively used to investigate cell behaviour *in vitro*. A well recognised potential difficulty with such "model" systems is that established cell lines, by virtue of either genetic or epigenetic adaptation to tissue culture conditions, may no longer resemble their alleged counterparts *in vivo*. Workers wishing to avoid such problems are therefore faced with the often daunting task of isolating and culturing homogeneous populations of cells from tissue samples containing a heterogeneous collection of cell types. Clearly, a variety of experimental approaches are required to deal with the particular problems presented by different types of tissue. The above text consists of a collection of articles, some of which are concerned with the application of specific techniques (eg. centrifugal elutriation, density gradient electrophoresis). In general, these articles are informative and will be of interest to a wide range of readers. The majority of articles in the text are concerned with techniques which may be used to prepare homogeneous cell populations from specific tissues (eg mammary gland, pancreatic islets). These articles also present information regarding the subsequent culture of cell types which commonly display rather stringent growth requirements *in vitro*. The authors have chosen well-recognised authorities in their respective fields to write the individual articles. I feel that the text will be a welcome addition to a departmental or laboratory library and will prove to be of considerable practical assistance to research workers grappling with these difficult experimental problems.

S. Schor,
Dept. Medical Oncology,
Christie Hospital & Holt Radium Institute,
Manchester.